# Obesity and psychotropic medication: a prospective register linkage study among midlife women and men

**DOI:** 10.1186/s12888-016-0889-3

**Published:** 2016-06-06

**Authors:** Anna Svärd, Jouni Lahti, Ossi Rahkonen, Eero Lahelma, Tea Lallukka

**Affiliations:** Department of Public Health, Faculty of Medicine, University of Helsinki, Tukholmankatu 8B, P.O. Box 20, 00014 Helsinki, Finland; Finnish Institute of Occupational Health, Helsinki, Finland

**Keywords:** Obesity, Overweight, Body mass index, Psychotropic medication, Mental ill-health, Register data, Follow-up, Ageing employee

## Abstract

**Background:**

Both obesity and mental health are major public health issues. This study aimed to examine whether overweight and obesity among midlife employees are associated with subsequent psychotropic medication. A further aim was to examine the potential effect of key covariates on the association.

**Methods:**

The Helsinki Health Study baseline survey was conducted in 2000–2002 among 40–60-year-old employees of the City of Helsinki, Finland (*n* = 8960). The participants were classified as of normal weight (18.5–24.9 kg/m^2^), overweight (25–29.9 kg/m^2^), obese (30–34.9 kg/m^2^) or severely obese (≥35 kg/m^2^) based on self-reported body mass index. Data on psychotropic medication purchases from baseline to 2009 were derived from registers of the Social Insurance Institution of Finland. The final analysis included 4760 women and 1338 men. Antidepressants and sedatives were examined separately. Covariates included socio-demographic factors, workload, health behaviours, physical functioning, somatic ill-health and psychotropic medication prior to baseline. Hazard ratios (HR) for the first psychotropic medication purchase were calculated using Cox regression analysis.

**Results:**

Third of women and quarter of men made at least one psychotropic medication purchase during the follow-up. Adjusting for age, obese (HR = 1.57; 95 % CI = 1.10–2.24) and severely obese (HR = 2.15; 95 % CI = 1.29–3.56) men were at risk of having psychotropic medication compared to men of normal weight. These associations disappeared after further adjustment. Severe obesity remained associated with subsequent sedative medication among the men even after full adjustment (HR = 2.12; 95 % CI = 1.17–3.84). No associations were found among the women.

**Conclusions:**

Obese and severely obese men, but not women, were at risk of psychotropic medication. Further studies are needed to deepen understanding of the relationship between obesity and mental ill-health, and the possible protecting effects of age, employment, and living environment.

## Background

Both obesity and mental ill-health are major public-health issues worldwide. In Finland, as in many other Western countries, one fifth of adults are obese and one tenth are regular users of psychotropic medication [1–4]. Previous studies have shown that both obesity and mental ill-health are likely independently to constitute risks for somatic diseases, poor quality of life, work disability and mortality [1, 5, 6].

The majority of relevant cross-sectional studies have shown an association between obesity and mental ill-health in the form of depression, anxiety, and suicide attempts and ideation among women [5–9], but some studies, especially among men, report no association [5–8]. Besides obesity also metabolic syndrome has been shown to be associated with mental ill-health [10]. The majority of longitudinal studies suggest that the association is reciprocal, supporting an association between obesity and subsequent mental ill-health [11–13]. It was shown in a meta-analysis that both overweight and obesity increase the risk of subsequent depression [11] and a review based on ten follow-up studies reported that in eight of them obesity was associated with subsequent depression [12]. However, in half of the reviewed studies the follow-up for obesity started in childhood or adolescence, in two of them the association disappeared following adjustment for baseline depression, and in one study on adolescents the association was found only among women [12]. The majority of the ten studies adjusted for sex, age, and education and half of the studies adjusted for marital status, somatic health, and smoking. However, only two studies adjusted for alcohol and three for physical activity.

In contrast with the majority of previous studies the most recent longitudinal studies report no or weak associations between obesity and subsequent mental ill-health [14–17]. According to a Finnish study, a high BMI among women, but not among men, predicted depressive symptoms, but the associations among women attenuated after adjustment for smoking and alcohol consumption [14]. In the largest follow-up study to date, obesity among female nurses in the US was found to predict self-reported depression or antidepressant use. The associations attenuated, but remained, following adjustments for baseline depression and age, comorbidities, health behaviours, and other covariates [13]. It was reported in another study among Baltimore adults that obesity did not increase the risk for depression or anxiety disorder, although an increased risk for suicide attempts was identified [14]. Similarly, a recent study using Mendelian randomisation reported no causal association between obesity and depression [16]. Longitudinal studies focusing on the association between obesity and anxiety disorder have also shown inconsistent results [6].

Our aim in this study was to provide a different perspective on the association and to find out whether baseline obesity among Finnish public-sector employees is associated with subsequent psychotropic medication. Only a few previous studies have focused on employed European adults, and many fail to adjust for earlier mental ill-health, socio-demographic and work- and health-related covariates. We considered age, socio-economic position, marital status, workload, smoking, alcohol consumption, physical activity, physical functioning, and somatic ill-health as key covariates as these factors are associated with mental ill-health and are often unequally distributed between the body mass index groups [18–21]. Moreover, given that most previous studies relied mainly on self-reported mental-health data, we offer a further perspective on the association between obesity and mental health by using register-based data on prescribed psychotropic medication. Our main aim was to investigate the potential association of overweight and obesity with subsequent psychotropic medication among midlife female and male employees. We also wanted to find out whether socio-demographic and work- and health-related covariates affected the associations, and whether there were differences between antidepressant and sedative medication.

## Methods

### Data

The Helsinki Health Study baseline mail surveys were conducted in 2000–2002 among 40–60-year-old employees of the City of Helsinki, Finland. There were 8960 respondents (response rate 67 %). Corresponding to the gender distribution in the Finnish municipal sector, 78 % of these respondents were women. Men, younger employees and manual workers were slightly underrepresented, but according to our non-response analyses, the data satisfactorily represent the target population [22]. Data on prescribed reimbursed psychotropic medication purchases from 1995 to 2009 were derived from registers of the Social Insurance Institution of Finland. The follow-up started from the day of returning the survey questionnaire and lasted until the end of 2009, the date of purchase of the psychotropic medication, or death (*n* = 176). The mean follow-up time was 6.4 years. The register data included the date of purchase, the type of prescribed medication and the amount in defined daily doses (DDD). Seventy-four per cent of the participants gave consent to the register linkages. According to our non-response analyses, employees from lower occupational and income classes were somewhat underrepresented, but the differences were small and are unlikely to cause a major bias in the results [23].

The 319 persons who were taking psychotropic medication at baseline were excluded. Further exclusions included 15 pregnant women, 58 underweight (BMI <18.5 kg/m^2^) individuals and 135 persons with missing information on some of the variables. Thus the final analysis included 6098 participants, of whom 78 % were women.

The ethics committees of the Department of Public Health, the University of Helsinki and the health authorities of the City of Helsinki approved the Helsinki Health Study protocol.

### Measures

#### Body mass index

We calculated the BMI (kg/m^2^) based on self-reported weight (kg) and height (m). We used the WHO (World Health Organization) recommendation in defining normal weight as 18.5–24.9 kg/m^2^, overweight as 25–29.9 kg/m^2^, obesity as 30–34.9 kg/m^2^ and severe obesity as ≥35 kg/m^2^.

#### Psychotropic medication

Prescribed, reimbursed psychotropic medication purchases, psychotropic medication in brief, was classified according to the Anatomical Therapeutic Chemical (ATC) system [24]. We included any psychotropic medication within the ATC classification N05 and N06 except anti-dementia medication (N06D), which we excluded. Antidepressants (N06A), sedatives (including anxiolytics [NO5B] and hypnotics and pure sedatives [NO5C]) were examined separately. The first prescribed, reimbursed psychotropic medication purchase during the follow-up implied psychotropic medication.

#### Covariates

The respondents were classified into five age groups based on age at baseline: 40, 45, 50, 55 and 60 years. Socioeconomic position was derived from the employer’s register and categorised into managers and professionals, semi-professionals, routine non-manual employees and manual workers [25]. Marital status included those who lived with a partner versus others. Workload was self-reported and categorised as physically and mentally strenuous or non-strenuous work. Leisure-time physical activity was measured in line with Metabolic Equivalent values (MET) [26], including four categories ranging from physically inactive to vigorously active [20]. Smoking at baseline was classified into current smokers and non-smokers. Drinking problems as measured on the CAGE questionnaire [27] included problem drinking and no problem drinking (the cut-off scores were two and three points for women and men, respectively). The physical functioning score from the Short-Form 36 questionnaire [28] was divided into quintiles, the lowest quintile representing poor physical functioning. Somatic ill-health was considered to be present if the respondent reported ever having been diagnosed with gout, arthrosis, rheumatoid arthritis, angina pectoris, myocardial infarction, cerebral circulation disturbance, claudication or epilepsy. Participants with at least one psychotropic medication purchase during a period of 5 years before baseline were categorized as prior psychotropic medication users. Participants who did not report problem drinking (*n* = 194), smoking (*n* = 36) or marital status (*n* = 26) were classified as not problem drinkers, non-smokers and singles, respectively. Respondents giving no information on workload (*n* = 99) were included in the non-strenuous category.

### Statistical methods

Cross-tabulation was initially used to describe the data. Kaplan-Meier curves were calculated to estimate the proportions of different weight groups with no psychotropic medication. Hazard ratios (HR) and 95-per-cent confidence intervals (95 % CI) for the first purchase of any psychotropic medication during the follow-up were calculated using Cox regression analysis. In addition, antidepressants and sedatives were examined separately. Normal weight was the reference category in all the analyses. Model 1 was adjusted for age and model 2 for age and previous psychotropic medication. Model 3 was also adjusted for covariates, including socio-economic position, marital status, physical and mental strenuousness of work, physical activity, smoking, and drinking behaviour. Model 4 was additionally adjusted for physical functioning and somatic ill-health. Women and men were examined separately. We used Schoenfeld’s residuals to confirm Cox proportional hazards assumptions. The analyses were performed using IBM SPSS Statistics 22.

## Results

**Fig. 1:**
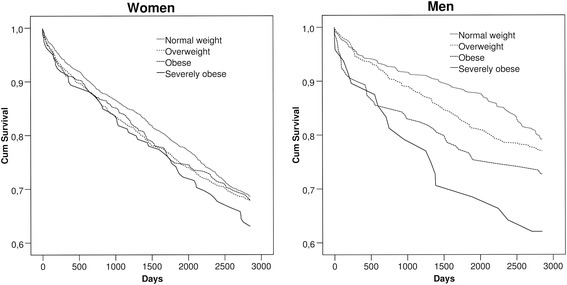
Estimated proportions of women and men with no psychotropic medication by BMI groups: Kaplan-Meier curves

Half of the women were of normal weight and a third were overweight (Table 1). About ten per cent of the women and the men were obese. As the Kaplan-Meier curves showed, obese and severely obese men were more likely than others to have had any psychotropic medication during the follow-up (Fig. 1). However, among the women the curves are descending at almost the same rate, suggesting only minor differences in psychotropic medication between the BMI groups.

**Table 1:** The distributions of the variables by BMI groups

	Body mass index (kg/m^2^)^a^									
	Women (*N* = 4760)					Men (*N* = 1338)				
	Normal weight	Overweight	Obese	Severely obese	Total	Normal weight	Overweight	Obese	Severely obese	Total
	%	%	%	%	%	%	%	%	%	%
Total	53	32	11	3	100	39	45	12	4	100
Age (years)										
40	25	17	17	14	21	22	18	11	4	18
45	24	20	18	19	22	22	20	15	21	20
50	22	22	20	21	22	19	20	20	38	20
55	21	26	32	32	24	26	27	37	23	28
60	9	15	14	14	12	12	16	19	15	15
Married/cohabiting	69	70	67	60	69	79	79	76	65	78
Socioeconomic position										
Managers/professionals	37	45	47	53	41	9	9	9	17	9
Semi-professionals	22	18	16	13	20	20	20	21	15	20
Routine non-manual	33	24	22	19	28	49	44	35	35	45
Manual workers	9	13	16	16	11	21	27	36	33	26
Physically strenuous work	34	41	43	44	38	16	14	17	19	15
Mentally strenuous work	75	76	77	74	76	74	75	70	71	74
Smoker	21	23	20	21	21	24	24	33	27	25
Drinking problem	15	16	14	14	15	22	23	32	19	23
Physical activity (MET)										
Inactive	18	25	38	48	24	20	25	44	56	26
Moderately active	38	42	41	34	40	33	32	31	25	32
Vigorously active	29	26	17	16	26	27	23	17	13	23
Highly active	15	8	4	2	11	21	20	8	6	18
Poor physical functioning	9	20	33	44	17	15	20	43	67	23
Somatic health problem	17	26	29	37	22	16	22	29	42	21
Psychotropic medication before baseline	19	23	26	21	21	11	14	21	25	14

^a^ Body mass index was categorised into four groups: normal weight 18.5–24.9 kg/m^2^, overweight 25–29.9 kg/m^2^, obese 30–34.9 kg/m^2^ and severely obese ≥35 kg/m^2^

A third of the women and a quarter of the men had taken some psychotropic medication during the follow-up (Table 2). The prevalence of antidepressant medication showed vague, but non-significant, trend to increase with increasing BMI among the women, whereas there was no such increase in the case of sedative medication. Among the men, psychotropic medication was more common among the obese (27 %) and the severely obese (38 %) than among those of normal weight (21 %). The prevalence of sedative medication increased with increasing BMI, being highest among the severely obese men (33 %).

**Table 2:** Psychotropic medication by BMI groups during the follow-up

	Body mass index (kg/m^2^)^a^					
	Normal weight	Overweight	Obese	Severely obese	Total	*P*-value^b^
	% (N)	% (N)	% (N)	% (N)	% (N)	
Women	54 (2545)	32 (1525)	11 (530)	3 (160)	100 (4760)	
Any psychotropic medication	31 (796)	32 (489)	32 (169)	36 (58)	32 (1512)	0.61
Antidepressants	21 (524)	22 (329)	23 (124)	25 (40)	21 (1017)	0.32
Sedatives	19 (493)	20 (300)	18 (95)	21 (33)	19 (921)	0.81
Men	39 (524)	45 (607)	12 (159)	4 (48)	100 (1338)	
Any psychotropic medication	21 (108)	23 (138)	27 (43)	38 (18)	23 (307)	<0.05
Antidepressants	15 (76)	16 (97)	18 (28)	23 (11)	16 (212)	0.41
Sedatives	12 (65)	15 (89)	18 (29)	33 (16)	15 (199)	<0.01

^a^ Body mass index was categorised into four groups: normal weight 18,5–24.9 kg/m^2^, overweight 25–29.9 kg/m^2^, obese 30–34.9 kg/m^2^ and severely obese ≥35 kg/m^2^

^b^ Chi-square test for proportions

We performed Cox regression analysis adjusting for covariates. Testing for gender interaction showed that the associations between BMI and any psychotropic medication (*p* = 0.04 for interaction) as well as sedatives (*p* = 0.01) were significantly different among the women and the men, whereas, for antidepressants the associations were similar (*p* = 0.44). Following adjustment for age, the obese (HR = 1.57; 95 % CI = 1.10–2.24) and severely obese (HR = 2.15; 95 % CI = 1.29–3.56) men were at risk of having some psychotropic medication during the follow-up compared to men of normal weight (model 1) (Table 3). The obesity risk weakened following adjustment for earlier psychotropic medication (model 2). Further adjustments in model 3 attenuated the risk for the severely obese (HR = 1.51; 95 % CI = 0.90–2.53). The association did not persist after additional adjustment for physical functioning and somatic health (model 4). When antidepressants and sedative medication were examined separately there were no associations for antidepressants among the men. However, obese (HR = 1.69; 95 % CI = 1.09–2.63) and severely obese (HR = 3.09; 95 % CI = 1.77–5.39) men were at risk of having sedative medication after adjustment for age. Further adjustment for previous psychotropic medication attenuated the association, which lost statistical significance among the obese. However, for the severely obese the association remained even after full adjustment (model 4) (HR = 2.12; 95 % CI = 1.17–3.84). No association was found among overweight men.

**Table 3:** The association of BMI with any subsequent psychotropic medication as well as antidepressant and sedative medication: Cox regression analysis (hazard ratios, HR, and their 95 % confidence intervals (95 % CI)

		Model 1		Model 2		Model 3		Model 4	
	BMI (kg/m^2^)^a^	HR	95 % CI	HR	95 % CI	HR	95 % CI	HR	95 % CI
Any psychotropic medication									
Women	Normal weight	1.00	-	1.00	-	1.00	-	1.00	-
	Overweight	1.06	0.95–1.19	1.00	0.89–1.12	0.97	0.87–1.09	0.94	0.84–1.06
	Obese	1.05	0.89–1.24	0.93	0.78–1.10	0.90	0.76–1.06	0.84	0.71–1.00
	Severely obese	1.23	0.94–1.61	1.23	0.94–1.61	1.16	0.89–1.53	1.03	0.79–1.36
Men	Normal weight	1.00	-	1.00	-	1.00	-	1.00	-
	Overweight	1.18	0.91–1.51	1.14	0.88–1.47	1.12	0.87–1.44	1.09	0.84–1.41
	Obese	1.57	1.10–2.24	1.37	0.96–1.97	1.26	0.87–1.82	1.15	0.79–1.66
	Severely obese	2.15	1.29–3.56	1.76	1.06–2.92	1.51	0.90–2.53	1.19	0.70–2.03
Antidepressants									
Women	Normal weight	1.00	-	1.00	-	1.00		1.00	-
	Overweight	1.10	0.96–1.26	1.05	0.91–1.20	1.00	0.86–1.15	0.96	0.83–1.11
	Obese	1.20	0.99–1.46	1.08	0.89–1.32	1.02	0.83–1.25	0.94	0.77–1.16
	Severely obese	1.32	0.95–1.82	1.29	0.93–1.77	1.15	0.83–1.60	1.01	0.72–1.40
Men	Normal weight	1.00	-	1.00	-	1.00	-	1.00	-
	Overweight	1.17	0.87–1.58	1.12	0.83–1.52	1.10	0.81–1.49	1.09	0.80–1.48
	Obese	1.44	0.93–2.22	1.20	0.77–1.86	1.05	0.67–1.65	0.95	0.60–1.50
	Severely obese	1.78	0.94–3.37	1.36	0.72–2.59	1.10	0.57–2.11	0.93	0.48–1.80
Sedatives									
Women	Normal weight	1.00	-	1.00	-	1.00	-	1.00	-
	Overweight	1.01	0.88–1.17	0.95	0.82–1.09	0.92	0.80–1.07	0.92	0.79–1.06
	Obese	0.91	0.73–1.13	0.79	0.64–0.99	0.78	0.62–0.98	0.76	0.61–0.95
	Severely obese	1.07	0.75–1.53	1.06	0.74–1.50	1.04	0.73–1.49	0.99	0.69–1.43
Men	Normal weight	1.00	-	1.00	-	1.00	-	1.00	-
	Overweight	1.26	0.91–1.73	1.26	0.91–1.73	1.27	0.92–1.75	1.21	0.87–1.68
	Obese	1.69	1.09–2.63	1.50	0.96–2.34	1.44	0.91–2.27	1.38	0.88–2.19
	Severely obese	3.09	1.77–5.39	2.61	1.49–4.55	2.44	1.38–4.31	2.12	1.17–3.84

^a^ Body mass index (BMI) was categorised into four groups: normal weight 18.5–24.9 kg/m^2^, overweight 25–29.9 kg/m^2^, obese 30–34.9 kg/m^2^ and severely obese ≥35 kg/m^2^

Model 1: Adjusted for age

Model 2: Adjusted for age and previous psychotropic medication

Model 3: Adjusted for covariates in model 2, socio-demographic factors, health behaviours and strenuousness of work

Model 4: Adjusted for covariates in model 3, physical functioning and somatic health

The associations between BMI and subsequent psychotropic medication were weak and non-significant among the women (Table 3). A weak association was found with antidepressants among the obese (HR = 1.20; 95 % CI = 0.99–1.46) and severely obese (HR = 1.32; 95 % CI = 0.95–1.82), but it disappeared after adjustment for the covariates.

## Discussion

### Principal findings

We examined the association between body mass index and subsequent psychotropic medication among midlife employees. Obesity and especially severe obesity were associated with having some psychotropic medication among the men, but the association attenuated following adjustment for previous psychotropic medication and other covariates. However, the association between severe obesity and sedatives among the men remained after adjustment. We found no association between BMI and subsequent psychotropic medication among the women.

### Previous studies

The majority of previous studies indicate an association between obesity and subsequent mental ill-health, findings that have been particularly consistent among women. We found no associations among the women, however, and only weak associations among the men. There are some differences to be noted when the results of this study based on prescribed, reimbursed psychotropic medication purchases are compared with earlier studies based mainly on self-reported mental-health data. First, our measure may be less sensitive to mental ill-health in that a medical prescription is based on a professional examination made by a physician in addition to symptoms reported by the patient. However, the threshold for prescribing psychotropic medication is probably quite low in Finland: the majority of common mental disorders are treated by general practitioners and access to reimbursed rehabilitative psychotherapy is restricted, and is first considered after 3 months of other appropriate treatment. Second, psychotropic medication for the obese may be affected by physicians’ preconceptions of obese people. It has been reported that physicians may consider obese people less motivated and compliant than patients of normal weight [29], and it is therefore possible that mental health among the obese is less likely to be treated as a medical problem. Third, obese people are more likely than those of normal weight to show other somatic health problems that require medical treatment, whereby psychotropic medication might be avoided due to the risk of drug interaction. Fourth, the threshold for consulting a doctor on account of mental ill-health might be higher among the obese than among those of normal weight. According to the results of one study, 84 % of obese participants agreed that “weight is blamed for most medical problems” [30]. However, the obese may have more regular contact with healthcare professionals on account of their somatic health, and thus their mental disorders could more easily be noticed.

In contrast to the majority of previous studies, the participants in our study were employed at baseline. There is one earlier study from the US that also focused on employees, but only female nurses were included and the participants were older, with an average age of 65 and most of them were probably no longer employed [13]. It is possible that work environments protect the obese from developing mental ill-health, given that the employed have been shown to have better health than the non-employed [31]. A recent study, using the same data, examined joint associations of BMI and leisure-time physical activity with psychotropic medication and suggested that physical activity dominates the association over normal-weight [32]. It is possible that employed people have healthier lifestyle e.g. less alcohol use and more exercise, which might partly protect them against mental ill-health. In further studies it would be interesting to compare employees with non-employed in order to study the differences in life style and the possible protective effect of employment.

Another difference from the majority of earlier studies is that our cohort focused on midlife people. Age has been discussed as both a protective and a risk factor for mental ill-health among the obese. It is suggested in a recent longitudinal study that the associations could be weaker among adults on account of improved emotional regulation and less stigmatisation associated with weight by age [15]. However, different views have also been presented, and a Finnish study has identified an association between advancing age and lower levels of body satisfaction on many aspects of body image [14, 33]. The study also showed that women associated their body mass index with their body image more strongly than men. It was therefore unexpected in our study that the association between obesity and mental ill-health was stronger among the men. However, the prevalence of psychotropic medication was higher among the women in our cohort, and it is possible that mental ill-health is perceived and treated differently among women and men, which might weaken the associations among women.

In contrast to the majority of earlier research, some recent studies [14–17] somewhat in line with our results, report no association between obesity and subsequent mental ill-health among women. It could be that the increased prevalence of obesity has changed people’s attitudes and made obesity more acceptable. Reducing the stigma attached to weight could positively affect self-esteem among the obese and protect against mental ill-health [34]. However, an extensive review did not produce evidence of a decrease in the stigmatisation of obesity [29], although it did show some differences between European countries compared with the US and Australia. Given such differences between countries, it would be interesting in further studies to focus on how the neighbourhood environment and culture contribute to the stigmatisation of weight and thus the association between obesity and mental health.

### Methodological considerations

The main strength of this study lies in the use of extensive prospective data (*n* = 6098) including employed women and men with different occupations and backgrounds. Another strength is the long follow-up time (a mean of 6.4 years). A third strong point is the use of reliable register data on prescribed psychotropic medication, including all prescribed reimbursed psychotropic medication purchased in pharmacies in Finland. Prescribed psychotropic medication from registers reflects mental health and provides data that avoids self-report bias. The register data on psychotropic medication have previously been used in order to examine associations between mental health and e.g. physical activity, workplace bullying, traffic noise, and marital status [20, 35–37].

However, our study population included only one, albeit large, municipal employer and is restricted to midlife employees. Height and weight measurements as well as the covariates are based on self-reports: people tend to underestimate their weight and to overestimate their height. However, self-reported BMI has been shown to predict sickness absence equally accurately as measured BMI [38]. The severely obese were few in number (*n* = 209), but the results did not differ from the ten per cent with the highest BMI. We also divided BMI into quartiles in the sensitivity analyses, instead of following the WHO recommendation, but this did not affect the results, either. The baseline-survey response rate was acceptable, but non-response remains a problem, and those under psychotropic medication may be overrepresented among the non-respondents.

A limitation with the data on medication is that the register does not include information on psychotropic medication that was prescribed but not purchased, or not reimbursed. We also lack information on the clinical indication of the prescribed medication. In some cases antidepressants are used to treat insomnia, chronic pain, premenstrual syndrome and anxiety disorder, whereas sedatives can be used during sudden life crises to treat insomnia or seizures, as well as anxiety. According to our sensitivity analyses, single prescriptions were evenly distributed over the weight groups, and even when 365 DDD was used as a cut-off point to indicate long-term psychotropic medication the results were similar. Given the overlapping of the clinical indication of the medication we decided to focus on psychotropic medication in general, and in addition examine antidepressants and sedatives separately. Because the indication of sedatives is varied, we carried out additional analyses separating anxiolytics (N05B) and pure sedatives (N05C, including sedatives and hypnotics). We found no association with anxiolytics among the men, but there was an association with pure sedatives. Sedative use could be related to sleep disorders such as obstructive sleep apnoea, which is a relatively common but frequently undiagnosed disorder among obese, middle-aged men [39]. Further studies are therefore needed to examine the association between psychotropic medication and sleep disorders, especially among obese men.

The Kaplan-Meier curves (Fig. 1) were steeper during the first year, suggesting a time-dependent association. However, when tested on the basis of Schoenfeld’s residuals the Cox proportional hazard assumptions held. Nonetheless, we carried out a sensitivity analysis in which the follow-up was cut off at 5 years, and the associations were only marginally stronger and again disappeared following adjustment. It is possible that adjusting for physical functioning and somatic health leads to over-adjustment in that physical health could also mediate the examined associations. For this reason we did not include physical health in model 3, although even when adjusted only for covariates in model 3, the statistical significance was lost.

## Conclusions

In conclusion, obesity and severe obesity were associated with subsequent psychotropic medication among the men, but the associations disappeared following adjustment for previous psychotropic medication and other covariates. However, the association between severe obesity and sedatives remained following adjustment. Obesity was not associated with subsequent psychotropic medication among the women. This calls for further studies to deepen understanding of the relationship between obesity and mental health, and to examine the possible protective effect of employment, age and the living environment. Further research is also needed to examine the indications and factors affecting the prescription of psychotropic medication.

## Abbreviations

ATC, Anatomical therapeutic chemical; BMI, Body mass index; CI, Confidence interval; DDD, Defined daily doses; HR, Hazard ratio; MET, Metabolic equivalent task; WHO, World Health Organization
